# Psychological and sociological determinants of entrepreneurial intentions and behaviors

**DOI:** 10.3389/fpsyg.2023.1076768

**Published:** 2023-02-02

**Authors:** Boštjan Antončič, Jasna Auer Antončič

**Affiliations:** ^1^School of Economics and Business, University of Ljubljana, Ljubljana, Slovenia; ^2^Faculty of Management, University of Primorska, Koper, Slovenia

**Keywords:** personality characteristics, sociological background, small and medium-sized enterprises, entrepreneur, entrepreneurship, start-ups

## Abstract

Research concerned with the personality of entrepreneurs entails an important part of the research into the management of small and medium-sized enterprises and entrepreneurship. This research has added new knowledge about the role of entrepreneurs’ personality characteristics, their family entrepreneurial background, and the local supportive entrepreneurial background in entrepreneurial start-up intentions and behaviors. Hypotheses and a model were developed and verified using structural equation modeling and regression analysis considering data from a sample of entrepreneurs and students. This research revealed that several personality and sociological factors can be important for entrepreneurship when it comes to starting a business. The most important were the Big Five personality factors openness, extraversion, and non-agreeableness and, to a smaller extent, emotional stability (non-neuroticism), and conscientiousness. The second-most important group of factors were the specific motivational characteristics entrepreneurial self-efficacy, internal locus of control, and risk-taking propensity. Sociological factors were much less important than psychological elements for establishing business.

## Introduction

Entrepreneurship is an individual-level or an organizational-level behavioral phenomenon and incorporates the creation and management of new businesses, small businesses, and family businesses, as well as the characteristics and special problems of entrepreneurs (Antončič, 2020). Research on the personality of managers of small and medium-sized enterprises (SMEs) and entrepreneurs is an important part of entrepreneurship and SMEs research. Views on entrepreneurs’ key personality characteristics are observable in research works in English (e.g., McClelland, 1961; Brockhaus, 1982; Baum et al., 2007; Rauch and Frese, 2007; Chell, 2008; Antončič et al., 2015; Salmony and Kanbach, 2022) and in other languages (e.g., in Slovenian: Petrin and Antončič, 1995; Antončič et al., 2002; Ruzzier et al., 2008). Alongside specific personality characteristics that have been primarily researched (e.g., need for achievement, internal locus of control, propensity to take risks, need for independence) and other approaches to specific personality characteristics (e.g., entrepreneurial self-efficacy), an approach to personality characteristics that is based on general personality characteristics (Big Five personality factors: openness, conscientiousness, extraversion, agreeableness, and neuroticism; Goldberg, 1981, 1990; Costa Jr. and McCrae, 1985) is seen as an area holding potential to connect personality characteristics with entrepreneurial activities (Singh and DeNoble, 2003; Antončič et al., 2008, 2015). After a comprehensive review of entrepreneurial personality, Baum et al. (2007) and Chell (2008) called for additional research into the personality of entrepreneurs and managers of SMEs. Rauch and Frese (2007) presented their previously developed model (Rauch and Frese, 2000) of the entrepreneur’s personality and success, which includes two groups of personality characteristics: broad personality characteristics (extraversion, emotional stability, openness to experience, agreeableness, and conscientiousness) and specific personality characteristics (need for achievement, risk-taking propensity, innovativeness, autonomy, locus of control, and self-efficacy). Rauch and Frese (2007) outlined the results of a meta-analysis on the relationship between personality characteristics and company success: broad (general) personality characteristics were related to success with *r* = 0.151, while specific personality characteristics were related to success with *r* = 0.231. Širec and Močnik (2010) discovered the partial consistency of the hypothesis about the connection of psychological motivational factors (need for achievement, risk-taking propensity, need for independence, self-image, self-efficacy, locus of control, and vision) with the growth of Slovenian companies. Antončič and Auer Antončič (2016) found a partial association of specific motivational personality characteristics (internal locus of control, entrepreneurial self-efficacy, need for achievement, need for independence, and propensity to take risks) of Slovenian entrepreneurs with the technological development and innovativeness of their companies. Salmony and Kanbach (2022) noted that personality traits (e.g., the Big Five, risk attitudes, locus of control, entrepreneurial self-efficacy, innovativeness, need for achievement) are crucial in entrepreneurship.

Research on entrepreneurs’ sociological characteristics is also an important part of the field of SME management and entrepreneurship. Shapero and Sokol (1982) emphasized that sociological and cultural factors may be important in the creation of entrepreneurial events and are most felt in the establishing of individual value systems. Business start-ups may depend on the presence of entrepreneurs as parents or siblings and on higher education (Dombrovsky and Welter, 2010). Schenkel et al. (2013) examined family entrepreneurial background and found no relationship with entrepreneurial intentions, although their study was limited by the use of a single-question measure to measure family entrepreneurial background. In this research, the association between family entrepreneurial background and entrepreneurship was assessed using a measure of family entrepreneurial background that includes various members of the family entrepreneurial environment (parents, grandparents, and siblings). Perceptions of the desirability and possibility of entrepreneurship in the entrepreneurial environment may be important for the establishment of firms (Krueger, 1993). Mentors and role models (Hisrich et al., 2013) and perceptions of opportunity and necessity (Reynolds et al., 2005; Wong et al., 2005) can also be important for a start-up and hence the characteristics of the local entrepreneurial background were also considered in this research.

Although there is evidence of a link between the Big Five personality traits and entrepreneurial intentions (e.g., Murugesan and Jayavelu, 2017; Şahin et al., 2019; Sahinidis et al., 2020) and entrepreneurship (intentions and behaviors, Antončič et al., 2015), the links between the Big Five personality traits of entrepreneurs, their family entrepreneurial background, and a local supportive entrepreneurial background, and business start-ups in a single model are lacking, so the past research did not identify, which of these personality or sociological elements could be more important for entrepreneurial intentions and behaviors in a model. This represents a gap in past research on SME management and entrepreneurship. The past research established that personality factors (e.g., Antončič et al., 2015; Murugesan and Jayavelu, 2017; Şahin et al., 2019; Sahinidis et al., 2020; Salmony and Kanbach, 2022), family entrepreneurial background (e.g., Dombrovsky and Welter, 2010; Schenkel et al., 2013; Georgescu and Herman, 2020), and the local supportive entrepreneurial background (e.g., Reynolds et al., 2005; Wong et al., 2005; Hisrich et al., 2013) are supportive elements to start a business, however from a theoretical and a practical viewpoint an examination of a relative importance of these factors for business start-up intentions and behaviors is lacking. In addition, Salmony and Kanbach (2022) pointed out a lack of research on the Big Five and entrepreneurship using actual entrepreneurs as participants. In this research, we added new knowledge about the role of personality characteristics of entrepreneurs, their family entrepreneurial background, and the local supportive entrepreneurial background in the establishing of companies. We thereby filled the gap in SME entrepreneurship and management research related to personality and sociological background in connection with the setting up of companies and expanded the research on personality and sociological background in entrepreneurship.

Salmony and Kanbach (2022) reviewed works on personality differences across different types of entrepreneurs and concluded that future studies need to conduct more systematic inquiries into the distinctions between sub-types of entrepreneurs and use evidently stated entrepreneurial samples. In this research, we examined effects of personality and sociological factors on entrepreneurship in terms of entrepreneurial intentions (persons with or without intentions to establish an enterprise) and entrepreneurial behaviors (actual entrepreneurs–founders and managers of SMEs).

## Theory and hypotheses

Work on developing a taxonomy of personality characteristics (Allport and Odbert, 1936; Cattell, 1943, 1945; Norman, 1967; Goldberg, 1981, 1990) led to the foundation of the Big Five factors with the initials OCEAN (Costa Jr. and McCrae, 1985), referring to the following (John, 1990, in Carducci, 1998, p. 239): the O factor: openness, originality, receptivity; the C factor: conscientiousness, control, constraint; the E factor: extroversion, energy, enthusiasm; the A factor: adaptability, agreeableness, altruism, adherence; the N factor: neuroticism, negative emotions, nervousness. The Big Five factors portray a relatively stable personality picture in adult persons (Schwaba and Bleidorn, 2018). Enterprises functioning mostly under the learning by doing-using-interacting mode can gain from owners’ Big Five personality characteristics (Runst and Thoma, 2022).

McClelland (1961) found that when we compare them with the population it is typical for entrepreneurs to not like repetitive and routine work, which can be classified as an openness factor. Several studies examining the entrepreneurship–openness relationship have determined that openness is a characteristic factor (Howard and Howard, 1995; Singh and DeNoble, 2003; Antončič et al., 2008, 2015). Openness of the entrepreneur and their creative personality may be important for the entrepreneur’s creativity and the growth of their firm (Peljko and Auer Antončič, 2022). Openness can be a very important factor for entrepreneurs because it plays a key role in the process of identifying an entrepreneurial opportunity. The tendency to act is a key element of entrepreneurship. Entrepreneurs pursue opportunities and turn ideas into profitable businesses. Identifying business opportunities may be considered one of the essential tasks that entrepreneurs are involved in during the entrepreneurial process, as well as the most fundamental task while beginning to create a new business. Opportunity recognition is accordingly the starting point of the entrepreneurial process (Baron, 2007). Discovery theory of entrepreneurial action assumes that entrepreneurs differ from non-entrepreneurs in their ability to see and exploit opportunities (Alvarez and Barney, 2007). The discovery and exploitation of opportunities are integral parts of the entrepreneurial process (Shane and Eckhardt, 2005). The openness factor can be the most important of the Big Five factors for distinguishing entrepreneurs from other individuals (Antončič et al., 2015):

*H1a*: A person’s openness is positively related to entrepreneurship in terms of starting a business.

McClelland (1961) found that entrepreneurs (compared to the population) score higher on need for achievement (the desire to do well). They take personal responsibility for their decisions, prefer decisions that involve moderate risk, dislike repetitive routine work, and are interested in concrete knowledge concerning the results of decisions. If we compare the content of these characteristics with the content of the Big Five factors, we can perceive the need for achievement as a personality characteristic of conscientiousness. The conscientiousness factor was shown not to separate between entrepreneurship-defining groups (Antončič et al., 2015), but Howard and Howard (1995) established that a high level of conscientiousness can be characteristic of an entrepreneurial type of person:

*H1b*: A person’s conscientiousness is positively related to entrepreneurship in terms of starting a business.

Howard and Howard (1995) determined that the entrepreneurial type can also be described as high in extraversion. A lower level of extraversion was found for non-entrepreneurs compared to entrepreneurs (Antončič et al., 2015):

*H1c*: A person’s extroversion is positively related to entrepreneurship in terms of starting a business.

Howard and Howard (1995) considered the entrepreneurial type to be average in agreeableness and a clear link between agreeableness and entrepreneurship thus cannot be expected. The dark side (Kets de Vries, 1985) could prevail, as seen in a study by Zhao et al. (2005) who reported that entrepreneurs had lower acceptance scores than managers. Agreeableness can be positively related to non-entrepreneurship (Antončič et al., 2015):

*H1d*: A person’s agreeableness is negatively related to entrepreneurship in terms of starting a business.

A personality characteristic of Western society may be unemotionality, which is important for personal success (Ryckman, 2000), suggesting the possibility of a negative association between neuroticism (the opposite of emotional stability) and entrepreneurship. Singh and DeNoble (2003) found a negative relationship between neuroticism and views of self-employment in terms of intention and perceived ability. Antončič et al. (2015) showed that the neuroticism factor might not distinguish entrepreneurs from non-entrepreneurs. The results of Goldberg (1990) support a possible negative neuroticism–entrepreneurship relationship because emotionally stable people are characterized by autonomy, independence, and individualism. Autonomy or independence may be related to entrepreneurship by serving as an important motivator (Collins and Moore, 1964; Licht and Siegel, 2006). Entrepreneurs can be somewhat neurotic (Lynn, 1969; Kets de Vries, 1977):

*H1e*: A person’s neuroticism is negatively related to entrepreneurship in terms of starting a business.

The forming of a new firm may depend decisively on family members as support persons and role models (Hisrich, 2013; Hisrich et al., 2013). The family business environment is important in entrepreneurship and can have effects on financial self-efficacy in certain economic milieus (Antončič et al., 2021). Lee et al. (2021) revealed that entrepreneurial family background of students strengthens the impact of entrepreneurship education on entrepreneurial passion for starting a company. Marques et al. (2018) studied entrepreneurship educations and discovered gender and family background variables as moderators with a positive impact on individual entrepreneurial orientation of students. Mitrovic Veljkovic et al. (2019) pointed out that family entrepreneurship background is important for entrepreneurial preferences of students. Georgescu and Herman (2020) found relationships between entrepreneurial family background, entrepreneurial personality traits, effectiveness of entrepreneurship education, and entrepreneurial intentions of students. One of the key driving elements of the sociological background for entrepreneurship can be family entrepreneurial experiences (Shapero and Sokol, 1982).

*H2a*: A person’s family entrepreneurial background is positively related to entrepreneurship in terms of starting a business.

The possibility and desirability of an entrepreneurial profession can be signaled through an entrepreneur’s environment (Shapero and Sokol, 1982; Antončič et al., 2002; Ruzzier et al., 2008; Hisrich et al., 2013). Entrepreneurial self-efficacy and entrepreneurial motivation can be developed based on community-level cultural norms (performance-based culture and socially supportive institutional norms) and can lead to the formation of new ventures (Hopp and Stephan, 2012). A person’s decision to become an entrepreneur can depend on a positive attitude to entrepreneurship (e.g., contact with entrepreneurs, the social desirability, regard, and reputation of entrepreneurs in society; Rebernik et al., 2014). Role models can impact entrepreneurial intentions and behaviors (Abbasianchavari and Moritz, 2021). Personal decisions about establishing a new firm can depend on friends, advisors, and support persons in the local neighborhood of an entrepreneur, and on a positive, opportunity-oriented, and encouraging environment (Hisrich, 2013; Hisrich et al., 2013).

*H2b*: A person’s local entrepreneurial support background is positively related to entrepreneurship in terms of starting a business.

## Research methods

### Participants

Data for this study were obtained through an online survey questionnaire. The data were collected from 366 entrepreneurs and non-entrepreneurs in Slovenia, namely electronically based on a representative sample of managers of Slovenian SMEs (128 usable answers) and in writing based on a purposeful sample of undergraduate business students at the School of Economics and Business of the University of Ljubljana (238 usable answers). Characteristics of participants are displayed in Table 1. Among the respondents, 33.6% were active entrepreneurs, 13.1% potential entrepreneurs, 41.5% possible entrepreneurs, and 11.7% non-entrepreneurs. By gender, there were slightly more women (51.4%) than men (48.6%), while there were fewer women among the managers. By age, there were more younger people (20 years or less 10.1%, over 20 to 30 years 55.5%, over 30 to 40 years 6.6%, over 40 to 50 years 10.7%, over 50 years 17.2%), whereas the managers were older than the students. Due to the differences between the two sub-samples, an additional control variable sub-sample (1–managers, 0–students) was introduced. Smaller companies dominated among the managers’ companies (number of employees by full-time equivalent: up to and including 10 66.4%, 11–50 28.1%, 51–250 5.5%; total sales in the previous year: EUR 400,000 or less 42.2%, over EUR 400,000 to EUR 800,000 18.0%, over EUR 800,000 to EUR 1,600,000 16.4%, over EUR 1,600,000 to EUR 4,000,000 14.8%, over EUR 4,000,000 8.6%), aged 11 to 50 years (82.9%), in service industries (81.9%; 18.1% were manufacturing companies).

**Table 1:** Characteristics of participants.

Characteristic		Frequency	Percent
Gender	Male	178	48.6
	Female	188	51.4
	Total	366	100.0
Age (in years)	20 or less	37	10.1
	Over 20 to 30	203	55.5
	Over 30 to 40	24	6.6
	Over 40 to 50	39	10.7
	Over 50	63	17.2
	Total	366	100.0
Status/base	Student	238	65.0
	SME manager	128	35.0
	Total	366	100.0

### Instrument

The model’s elements and questions for the measurement questionnaire were conceptually developed mainly based on questions from past research. First, general personality characteristics (the Big Five factors) were assessed as measured by Singh and DeNoble (2003) and Antončič et al. (2008, 2015), who used Saucier’s (1994) Mini-Markers Inventory, which includes 8 adjectives for each personality factor: (1) openness–adjectives: creative, imaginative, philosophical, intellectual, complex, deep, non-creative (r), non-intellectual (r); (2) conscientiousness–adjectives: organized, efficient, systematic, practical, disorganized (r), sloppy (r), inefficient (r), carefree (r); (3) extraversion–adjectives: talkative, extroverted, bold, energetic, reserved (r), quiet (r), shy (r), introverted (r); (4) agreeableness–adjectives: sympathetic, warm, friendly, cooperative, cold (r), unsympathetic (r), rough (r), strict (r); (5) neuroticism–adjectives: unenvious (r), relaxed (r), capricious, jealous, temperamental, envious, sensitive, irritable. Respondents were asked about their level of agreement with 40 adjectives on a Likert-type scale with anchors ranging from 1–does not apply very much to 5–applies very much. The Big Five personality factors showed a satisfactory to very good level of reliability (Cronbach’s alpha: Openness 0.74, Conscientiousness 0.77, Extraversion 0.81, Agreeableness 0.73, Neuroticism 0.66–two questions eliminated: non-envious, relaxed).

Second, the sociological background was measured with 17 questions on a Likert-type scale with anchors ranging from 1–strongly does not apply to 5–strongly applies. Seven questions measured family entrepreneurial background: my father is or was an entrepreneur, my mother is or was an entrepreneur, my grandparents are or were entrepreneurs, my great-grandparents and/or their ancestors were entrepreneurs, my brothers or sisters are or were entrepreneurs, there are many entrepreneurs in my extended family, and I was brought up in an environment of family entrepreneurship. Ten questions measured the local entrepreneurial support background: I personally know many entrepreneurs, my friends are entrepreneurs, my advisors are entrepreneurs, my role models are entrepreneurs, I grew up in a neighborhood with a large number of entrepreneurs, I grew up in a neighborhood that was very supportive of entrepreneurs, I grew up in a neighborhood that forced individuals into entrepreneurship, I grew up in an environment where entrepreneurship was seen as an opportunity, I grew up in an environment where entrepreneurship was seen as a necessity, and I grew up in a positive environment for entrepreneurship. The level of reliability was very good for family entrepreneurial background (Cronbach’s alpha 0.83) and local entrepreneurial support background (Cronbach’s alpha 0.81).

The final dependent variable–entrepreneurship (purposes and activities) in terms of starting a business–was assessed with the measure from Antončič et al. (2007): entrepreneurs (actual business), potential entrepreneurs (intention to start a business in the next 3 years), possible entrepreneurs (who could start a business in the future), and non-entrepreneurs (who do not intend to start a business). Entrepreneurship variables are shown in Table 2. The first variable was designed based on this classification of business creations or entrepreneurship in four ascending classes (1–non-entrepreneurs, 2–possible entrepreneurs, 3–potential entrepreneurs, and 4–actual entrepreneurs). The second variable was designed to distinguish actual entrepreneurs (1) from others (0). Potential entrepreneurs are usually more similar to actual entrepreneurs (Antončič et al., 2015) and hence, the third variable was coded as 1–entrepreneurs (actual and potential) and 0–non-entrepreneurs (possible entrepreneurs and non-entrepreneurs). In the structural modeling, the third variable was primarily used and, where possible, a construct consisting of all three variables was also used. In the regression analysis, a summary variable (arithmetic mean calculated based on the second and third variables).

**Table 2:** Entrepreneurship variables.

Varable		Frequency	Percent
Entrepreneurship 1	1: No intention (non-entrepreneur)	43	11.7
	2: Low intention (maybe-entrepreneur)	152	41.5
	3: High intention (potential entrepreneur)	48	13.1
	4: Behavior (actual entrepreneur)	123	33.6
	Total	366	100.0
Entrepreneurship 2	0: Non-entrepreneur (no enterprise)	243	66.4
	1: Entrepreneur (enterprise)	123	33.6
	Total	366	100.0
Entrepreneurship 3	0: Non-entrepreneur (no/low intention)	195	53.3
	1: Entrepreneur (potential/actual)	171	46.7
	Total	366	100.0

Measures of specific motivational personality characteristics and other control variables were also included in the questionnaire. Alongside the Big Five, the key personality correlates of entrepreneurship comprise specific personality characteristics (Antončič, 2020): internal locus of control, entrepreneurial self-efficacy, need for achievement, need for independence, and risk-taking propensity, so they were included as control variables. Specific motivational personality characteristics were measured with the following questions (on a Likert-type scale with anchors from 1–very much not true to 5–very much true): Internal locus of control included a question, I have control over my destiny, and five questions from Chen et al. (1998): I can usually protect my personal interests; my life is determined by my own actions; I can pretty much determine what will happen in my life; what I plan I almost certainly make work; when I get what I want, it’s usually because I worked really hard for it. The level of reliability of the internal locus of the control construct was satisfactory (Cronbach’s alpha 0.69). Entrepreneurial self-efficacy consisted of five questions: I am capable of successfully implementing marketing; I am capable of successful implementation of innovations; I am capable of successful implementation of management; I am capable of successfully taking risks; I am able to successfully implement financial control. These five questions are consistent with the definition of entrepreneurial self-efficacy given by Chen et al. (1998), yet also contain fewer questions than the measure of these authors. The level of reliability of the entrepreneurial self-efficacy construct was very good (Cronbach’s alpha 0.81). The need for achievement was measured by one question: I have a desire for achievement, from Antončič and Auer Antončič (2011). The need for independence entailed one question: I have a desire for personal independence, from Gantar et al. (2013). Risk-taking propensity was measured with two questions from Auer Antončič et al. (2018a): I like to take risks; I am risk-averse. The two risk-taking propensity questions were highly correlated (Pearson’s correlation coefficient 0.71, sig. 0.000). Measures for other control variables (person-related: gender and age; company-related: industry, age, and size) were assessed following Auer Antončič et al. (2018b).

### Procedure

The data analysis was quantitative. The constructs were verified by exploratory (tool used: SPSS) and confirmatory factor analysis (tool: EQS). The hypotheses and the model were verified using structural equation modeling (tool: EQS) and regression analysis (tool: SPSS). Structural equation modeling used latent factors determined based on measurement variables for each construct. In the regression analysis, the variables were calculated as arithmetic means of the measurement variables for each construct.

Common method bias was verified *via* Harman’s (1976) one-factor test, which did not indicate a presence of common method bias (the total variance explained by a single factor was 16.2%, which is well under the 50% threshold of Podsakoff and Organ, 1986).

## Empirical results

### Structural equation modelling results

**Figure 1:**
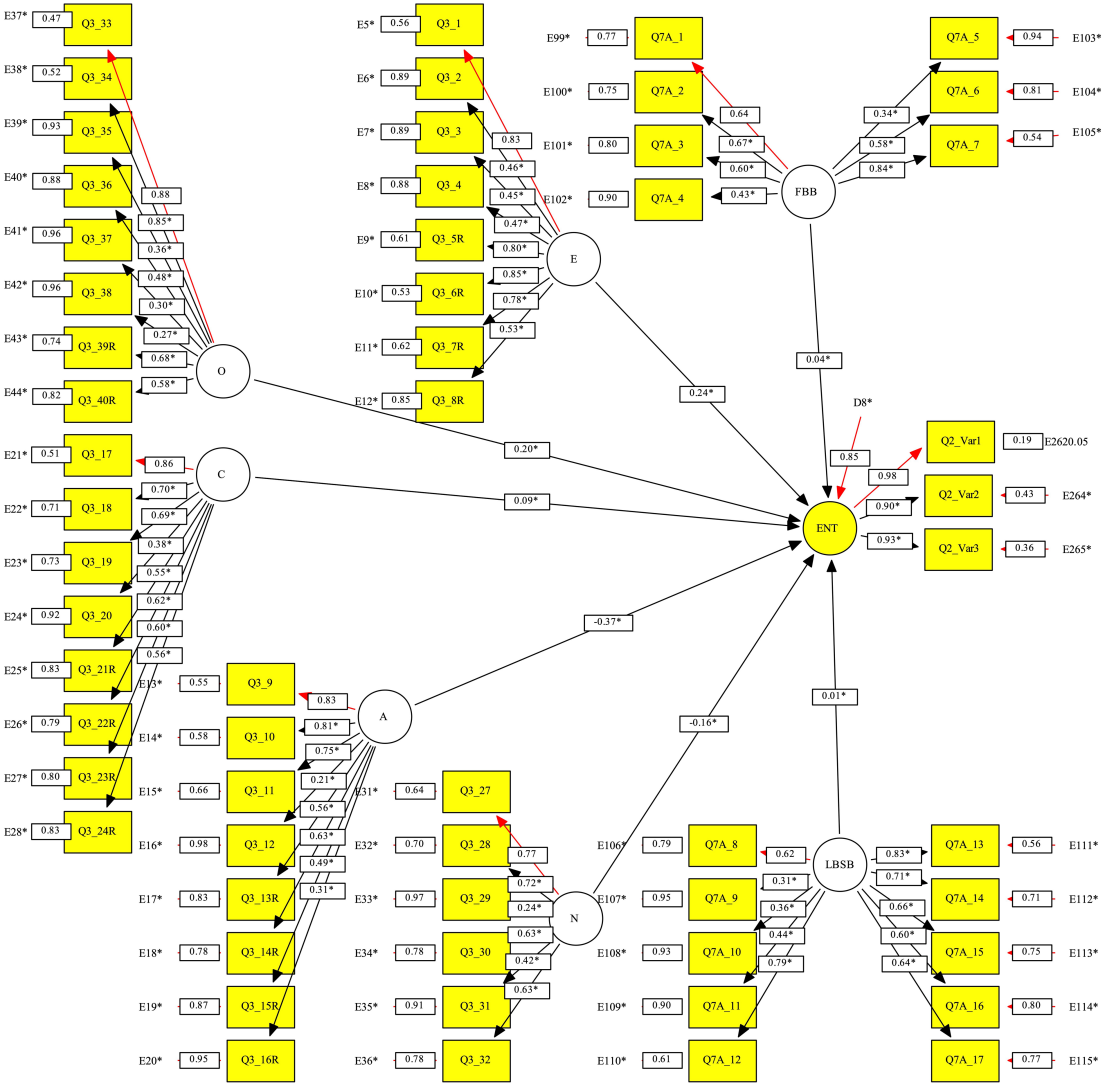
The model of the Big Five factors of personality, sociological entrepreneurial background, and establishment of companies (structural equation modeling, standardized solution). ENT, Entrepreneurship in terms of starting a business; O, Openness; C, Conscientiousness; E, Extraversion; A, Agreeableness; N, Neuroticism; FBB, Family business background; LBSB, Local business support background; Qx, items; Ex, errors; D, disturbance; *, estimated parameters.

We tested a structural equation model that included the Big Five personality factors and two sociological entrepreneurial environment factors as independent factors and entrepreneurship in terms of starting a business as a dependent factor. Before the structural equation model was estimated, each construct was tested by using exploratory and confirmatory factor analysis, which indicated adequate results in terms of convergent validity, discriminant validity, composite reliability, and factor loadings (positive and significant). The structural equation model is shown in Figure 1. The model was checked using the data of 295 persons (we skipped 71 out of 366 due to them missing at least one item in the response). The model had a very good fit and reliability (NFI 0.97, CFI 0.98, RMSEA 0.075, and Cronbach’s alpha 0.81). It predicted 27.2% of the variance in the dependent factor of business start-up (determined by the three start-up variables). The statistically significant standardized coefficients shown in Table 3 are consistent with the hypotheses for four personality factors (openness, H1a; extraversion, H1c; agreeableness, H1d; neuroticism, H1e), and statistically insignificant, but in the right direction, for one personality factor (conscientiousness, H1b) and two factors of entrepreneurial sociological background (family entrepreneurial background, H2a; local entrepreneurial support background, H2b).

**Table 3:** The model of the big five factors of personality, sociological entrepreneurial background, and establishment of companies (structural equation modeling).

Independent factor	Standardized coefficient
Openness	0.198*
Conscientiousness	0.088
Extraversion	0.245*
Agreeableness	−0.372*
Neuroticism	−0.158*
Family business background	0.038
Local business support background	0.015

Dependent factor: Entrepreneurship in terms of starting a business. Coefficient of determination: 0.272. *p < 0.05.

By introducing person-level control variables (specific motivational personality characteristics) in a regression model predicting business start-ups without sociological factors, two were found to be statistically significant (entrepreneurial self-efficacy, need for achievement) and positively related to business start-ups. The proportion of explained variance in the dependent variable of business creation was significant (14.7%), but not considerably higher than in the model without these control variables. Here, the connections between openness and founding and neuroticism and founding became statistically less significant (sig. < 0.10) compared to the results in Table 3.

### Regression analysis results

The multiple regression analysis supported the results of the structural equation modeling. The regression model predicted 14.0% of the variance in the dependent factor of business start-up (determined by the average start-up variable). The statistically significant standardized coefficients presented in Table 4 are consistent with the hypotheses for four personality factors (openness, H1a; extraversion, H1c; agreeableness, H1d; neuroticism, H1e), and one entrepreneurial sociological background factor (local entrepreneurial support background, H2b), and are statistically non-significant, but in the right direction, for one personality factor (conscientiousness, H1b), whereas statistically non-significant and not in the right direction for one factor of entrepreneurial sociological background (family entrepreneurial background, H2a).

**Table 4:** The model of the big five factors of personality, sociological entrepreneurial background, and establishment of companies (multiple regression analysis).

Independent variable	Standardized coefficient
Openness	0.119*
Conscientiousness	0.065
Extraversion	0.160*
Agreeableness	−0.214*
Neuroticism	−0.112*
Family business background	−0.064
Local business support background	0.150*

Dependent variable: Entrepreneurship in terms of starting a business. Coefficient of determination: 0.140. *p < 0.05.

By introducing person-level control variables (gender and age) in a regression model predicting business start-ups, both were determined to be statistically significant (age positively related: older people related to start-ups more than younger people; gender: men related to start-ups more than women). The proportion of explained variance in the dependent variable of business establishment was high (62.4%), also indicating a strong effect of age and gender. Here, the connections between openness and start-ups and neuroticism and start-ups became statistically non-significant, while the connection between family entrepreneurial background and start-ups became statistically significant compared to the results in Table 4.

We also performed simple regression analyses and correlation analyses (results identical in content) to identify bivariate associations between variables that were obscured (reduced levels of association) due to multicollinearity. Statistically significant relationships of entrepreneurship in terms of starting a business were found with the following variables (correlation coefficients in parentheses): openness (0.211), conscientiousness (0.156), extraversion (0.235), agreeableness (−0.164), entrepreneurial self-efficacy (0.168), internal locus of control (0.243), risk-taking propensity (0.110), gender (0.747), industry (−0.390), total sales in the past year (−0.259), base (0.800). These results reveal a bivariate association of four of the five personality factors (consistent with hypotheses H1a, H1b, H1c, and H1d) and several control variables with the establishment of a business.

## Discussion, contributions, and implications

This research showed that several personality and sociological factors can be important for entrepreneurship when it comes to starting a business. First, the most important were the Big Five personality factors openness, extraversion, and non-agreeableness, and to a smaller extent emotional stability (non-neuroticism) and conscientiousness. These results for openness, extraversion, and non-agreeableness are in line with findings of Antončič et al. (2015) and partially comparable to their findings for conscientiousness and neuroticism, because Antončič et al. (2015) showed that the conscientiousness factor and the neuroticism factor might not distinguish entrepreneurs from non-entrepreneurs.

The second-most important factors were the specific motivational characteristics entrepreneurial self-efficacy, internal locus of control, and risk-taking propensity. These results are: (1) congruent with past studies (e.g., Antončič, 2020; Salmony and Kanbach, 2022) for the three characteristics (entrepreneurial self-efficacy, internal locus of control, and risk-taking propensity); and (2) contradictory to Salmony and Kanbach (2022) for the need for achievement, and to Antončič (2020) for the need for achievement and the need for independence. The use of different specific motivational characteristics in this study, enabled us to find, which specific characteristics can be more important than other characteristics. These peculiar results warrant further investigation in future studies.

Third, sociological factors were much less important than psychological elements for establishing a business, yet entrepreneurial local support background showed some effect. Interestingly, the finding that a person’s family entrepreneurial background is not related to entrepreneurship (in terms of starting a business) contradicts findings of past studies based on students (e.g., Marques et al., 2018; Mitrovic Veljkovic et al., 2019; Georgescu and Herman, 2020; Lee et al., 2021) and confirms the notion that actual entrepreneurs need to be included as respondents in entrepreneurship research (Salmony and Kanbach, 2022).

Fourth, a person’s age (and the role of an entrepreneur vs. a student) and gender, and a company’s industry and sales were shown as essential control variables related to business start-ups. The person’s age result (age positively related to entrepreneurship: older people related to start-ups more than younger people) contradicts past studies (e.g., Krueger and Brazeal, 1994; Levesque and Minniti, 2006; Singh, 2014), which indicated a negative relationship (younger persons should have higher entrepreneurial intention than older persons). The person’s age result of our study may be related to the characteristics of the sample: students were younger and less entrepreneurial than SME managers. The positive relationship between gender and entrepreneurship (men related to start-ups more than women) is aligned with past research (e.g., Shinnar et al., 2012; Strobl et al., 2012; Singh, 2014; Antončič et al., 2015). The results related to company-level controls (industry and sales) may be less revealing because they are based solely on the sub-sample of SME managers.

The main scientific contribution of this research is the theoretically developed and empirically verified new model containing personality characteristics and the characteristics of the sociological background that encouraged business start-ups, which includes personality variables (Big Five personality factors), family entrepreneurial and local support background variables, and specific personality and demographic control variables. Following the suggestions of Antončič et al. (2015) and somewhat less so the findings of Rauch and Frese (2000, 2007), we found that the Big Five are the most important for starting companies, with specific motivational personality characteristics also being important, and sociological factors being less important. This study contributes to past research on entrepreneurial personality (e.g., Antončič et al., 2015; Murugesan and Jayavelu, 2017; Şahin et al., 2019; Sahinidis et al., 2020; Salmony and Kanbach, 2022) and sociological determinants of entrepreneurship (family entrepreneurial background, e.g., Dombrovsky and Welter, 2010; Schenkel et al., 2013; Marques et al., 2018; Mitrovic Veljkovic et al., 2019; Georgescu and Herman, 2020; Lee et al., 2021; local supportive entrepreneurial background, e.g., Reynolds et al., 2005; Wong et al., 2005; Hisrich et al., 2013) by developing the model and providing evidence about the relative importance of psychological and sociological factors for business start-up intentions and behaviors. This study contributes in terms of methodology to research on the Big Five personality characteristics and entrepreneurship (e.g., Murugesan and Jayavelu, 2017; Şahin et al., 2019; Sahinidis et al., 2020; Salmony and Kanbach, 2022) by using a sub-sample of actual entrepreneurs and three variables of entrepreneurship. The research has implications for theory. Entrepreneurship researchers should include both psychological and sociological variables in their models, use control variables (age and gender), various entrepreneurship variables, and samples or sub-samples of actual entrepreneurs.

The research holds implications for practice. Suggestions for people who would like to commence a business: Starting a business should be a challenge for both younger and older people. The elderly should not feel too old to start a business because they can be a rich source of knowledge and experience. Younger people should accept the support of older people in starting a company, or young people who can be motivated and full of work zeal should also start a company. It is recommended that older people help them in this and in so doing combine knowledge, work experience, and drive since research shows that older people are more connected to start-ups than younger ones. Companies should also be founded by women, despite the results of this research revealing that men are more associated with establishing them than women.

We recommend the establishment of a company primarily to open, extroverted, and less agreeable individuals, and for an easier entrepreneurial start for those who are not we suggest that they connect with open, extroverted, and less agreeable people when establishing and making the main decisions while managing the company, as it has been shown that openness, extroversion, and non-agreeableness can be important for starting a business. If a person likes new things, is creative, original, imaginative, innovative, and eager for change, they will most likely know what they want to do, they will be sure that they want to realize their ideas, and it will be easier to become an entrepreneur. New ideas and alternatives can enable diversity and business expansion. For the founder of a company, it is very important that they are open as a person; namely, intellectual and complex. An individual could even improve their personality characteristics to some extent through training and coaching because a person’s Big Five personality characteristics are partly learned and partly innate (Antončič, 2009; Auer Antončič, 2012).

A person who is lively, full of energy, active, cheerful, dominant, bold, and unwavering will most likely establish contact with the outside public more easily and thus deal with the establishment of the company’s operations more easily and quickly. For persons who are communicative, sociable, ambitious, determined, spontaneous, adventurous, cheerful, connecting, and open to new people, it is easier when launching a company, most likely due to their larger friendship and inter-organizational networks which may provide support during its establishment. Moreover, emotionally stable people may like to consider starting their companies, i.e., those people who do not become angry easily, are rarely irritable and envious, such that there will perhaps be less conflict and stressful situations at the beginning of the entrepreneurial journey. Other sub-dimensions of neuroticism like anger, depression, and personal anxiety can also cause additional problems, grievances, non-cooperation, disagreement, and disloyalty among colleagues while setting up a company (Auer Antončič, 2012).

A less lenient individual might be more successful in starting a business because with agreeableness as a personality trait a person can quickly change their decisions and not stick to agreements. The goals of less agreeable people can be more in the foreground and individuals can realize their vision more easily (Auer Antončič, 2012).

Launching a new idea or a new unit should also be tried by individuals who come from a local environment favorable to entrepreneurship since this background appears to be somewhat important for the establishment of a company.

The research has implications for the economy and society. Proposals on the level of the whole economy: Economic policymakers should strive to promote the factors that contribute to the establishment of enterprises through various mechanisms. Above all, it is necessary to encourage and develop openness in people given that openness can be important for the setting up of a company. It would also make sense to develop extroversion since that can be important for establishing companies. In addition, non-adherence could be encouraged as it may be important while establishing companies. This last suggestion regarding non-agreeableness may seem unusual or contrary to social norms as it would encourage characteristics like non-sympathy, indifference, unfriendliness, uncooperativeness, coldness, rudeness, and strictness, and we should hence be very careful with it. We suggest that those designing the education system devote themselves to the design of educational programs and content that will promote personality development, especially openness (e.g., creativity and imagination and philosophical, intellectual, and deep thinking) and extraversion (e.g., eloquence, boldness, energy, unrestrained thinking, and shamelessness) and possibly emphasize the positive role of the sociological factor of the local business environment.

## Limitations and future research possibilities

There are some limitations of this research, e.g., in terms of the number of factors, the sample, and the data collection. The research is limited to the personality characteristics of persons and the sociological entrepreneurial background as an important set of factors that can contribute to the establishment of companies. Other factors that might be important for the setting up of companies (e.g., innovativeness, Alshebami and Seraj, 2022; creativity, Peljko and Auer Antončič, 2022; narcissism and resilience, Leonelli et al., 2022; improvisation, Guo et al., 2022; emotional intelligence, Lopez-Nunez et al., 2022; epistemic curiosity and entrepreneurial alertness, Heinemann et al., 2022; financial rewards and social recognition, Ismail, 2022; theory of planned behavior components: personal attitude, subjective norms, and perceived behavioral control, Maheshwari, 2021) were not included in the research and can be added in future studies.

Due to the limitation of the sample, the respondents were selected only in Slovenia, although the results of the research could also be transferred to other countries through future comparative studies. In future cross-national and/or cross-cultural studies, additional sociological factors reflecting cross-cultural differences may reveal impacts in the model. The SME sample is representative since it was collected through random sampling, whereas the student sample is purposive. In future research, it would be reasonable to use representative samples of persons on the population level.

The data were obtained based on a questionnaire that mainly had closed-type questions for later accurate data processing. In the questionnaire, an individual’s subjective attitudes regarding individual claims were checked. The individual answered questions or statements by choosing from already given answers or statements, which may be a disadvantage, on the one hand, because there are usually only a limited number of such statements. Such predetermined statements, on the other hand, can provide an advantage because they are less likely to elicit ambiguous or overly broad responses from respondents. The most suitable method of data collection for this quantitative research was a closed-ended questionnaire, which allowed us to know all possible answers with sufficient reliability and that there were not too many of them. Future qualitative research could illuminate and expand our knowledge of the factors discussed. For example, a future qualitative study can examine in depth the meaning of psychological and sociological aspects of the entrepreneurial decision.

## Conclusion

This research contributed a new model containing personality characteristics and characteristics of the sociological background that encouraged business start-ups (intentions and behaviors), which includes personality variables (Big Five personality factors), family entrepreneurial, and local support background variables, as well as control variables. Future research should explore and supplement this model in greater detail.

## Data availability statement

The datasets presented in this article will be made available by the authors upon request. Requests to access the datasets should be directed to bostjan.antoncic@ef.uni-lj.si; jasna.auer@fm-kp.si.

## Ethics statement

Ethical review and approval was not required for the study on human participants in accordance with the local legislation and institutional requirements. Written informed consent for participation was not required for this study in accordance with the national legislation and the institutional requirements.

## Author contributions

BA and JA developed the research project, carried out the data collection and analysis, wrote the first draft, and revised the manuscript. All authors contributed to the article and approved the submitted version.

## Funding

This work was supported (e.g., grants for data collection, research work, and open-access publication fees) by the Slovenian Research Agency (project/grant numbers P5-0117 and J5-7588) and the University of Ljubljana, School of Economics and Business (project/grant IRD and IRS).

## Conflict of interest

The authors declare that the research was conducted in the absence of any commercial or financial relationships that could be construed as a potential conflict of interest.

## Publisher’s note

All claims expressed in this article are solely those of the authors and do not necessarily represent those of their affiliated organizations, or those of the publisher, the editors and the reviewers. Any product that may be evaluated in this article, or claim that may be made by its manufacturer, is not guaranteed or endorsed by the publisher.
